# Determinants of macular ganglion cell–inner plexiform layer thickness in normal Chinese adults

**DOI:** 10.1186/s12886-021-02023-0

**Published:** 2021-06-29

**Authors:** Xiaoyu Xu, Hui Xiao, Kunbei Lai, Xinxing Guo, Jingyi Luo, Xing Liu

**Affiliations:** 1 State Key Laboratory of Ophthalmology, Zhongshan Ophthalmic Center, Sun Yat-sen University, 7 Jinsui Road, Tianhe District, Guangzhou, Guangdong PR China 510623; 2 Wilmer Eye Institute, John Hopkins University School of Medicine, Baltimore, MD USA

**Keywords:** Ganglion cell–inner plexiform layer, Determinants, Optical coherence tomography, Normal, Adults

## Abstract

**Background:**

Demographic, systemic and ocular factors may impact macular ganglion cell–inner plexiform layer (GCIPL) thickness measurements. This study aimed to investigate the influences of multiple potential determinants of macular GCIPL thickness in normal Chinese adults.

**Methods:**

This was a retrospective study conducted on 225 normal eyes from 225 healthy Chinese adults. GCIPL thickness were obtained using Cirrus high-definition optical coherence tomography (OCT). The age, gender, laterality, spherical equivalent (SE) refractive error, intraocular pressure (IOP), axial length (AL), central cornea thickness (CCT), circumpapillary retinal nerve fibre layer (pRNFL) thickness and OCT signal strength were recorded and their respective effect on GCIPL thickness parameters were evaluated.

**Results:**

The mean (± SD) average, minimum, superotemporal, superior, superonasal, inferonasal, inferior, and inferotemporal GCIPL thickness was 84.56 ± 5.36, 81.32 ± 5.58, 83.08 ± 5.37, 85.70 ± 5.95, 87.15 ± 6.26, 85.07 ± 6.11, 82.46 ± 5.76, and 83.88 ± 5.59 μm, respectively. Determinants of thinner GCIPL thickness were older age (*P* = 0.001–0.117; effects enhanced if age over 40 years), thinner pRNFL (all *P* < 0.001), and weaker signal strength (all *P* < 0.001). No significant difference was found between males and females (*P* = 0.069–0.842), and between right eyes and the left eyes (*P* = 0.160–0.875) except that of superonasal GCIPL thickness (*P* < 0.001). There was no significant correlation between GCIPL thickness and SE, IOP, CCT, and AL (*P* = 0.135–0.968).

**Conclusions:**

Individual determinants associated with thinner GCIPL thickness were older age (particularly over 40 years of age), thinner pRNFL, and weaker OCT signal strength. This is relevant in comprehensively understanding the normative data and differentiating normal aging from abnormalities.

## Background

Glaucoma is one of the leading causes of irreversible blindness worldwide. The hallmark of glaucoma is progressive loss of retinal ganglion cells (RGCs) in the inner retina and their axons in the optic nerve head (ONH) [1]. Previous studies have showed that structural glaucomatous changes primarily affect RGCs and their axons, followed by functional changes featured by characteristic glaucomatous visual field defect [2]. There may be a 20–40% of RGC loss prior to detectable visual field defect, indicating that evaluation of RGCs could be beneficial in detection and monitoring of glaucoma [3].

As the output neurons of the retina, RGCs are the only part of the central nervous system that can be optically detected in vivo [4]. The human retina contains an estimate of more than 1 million RGCs, over 50% of which locate in the macular, making it the easiest region for calculating the RGC counts and detecting their loss [5]. The thinning of macular ganglion cell layer (GCL) and peripapillary retinal nerve fiber layer (pRNFL) has been well recognized as biomarkers of optic nerve damage, which can be quantitatively measured by optical coherence tomography (OCT) in a non-invasive, precise, and reproducible way.

A number of studies have showed that the macular ganglion cell complex (GCC, the sum of RNFL, GCL, and inner plexiform layer) thickness or the macular ganglion cell–inner plexiform layer (GCIPL) thickness has similar glaucoma discriminating performance with that of pRNFL [6–9]. Combining GCC/GCIPL thickness, pRNFL thickness, ONH parameters, psychophysics examinations, and vasculature evaluations, though, can help to improve the overall glaucomatous diagnostic ability and accuracy [10].

For clinical interpretation of GCC/GCIPL and pRNFL thickness readings, it is essential to know physiological factors that could influence these measurements. In normal eyes, age, sex, ethnicity, refractive power, axial length, optic disc size, OCT scanning mode and signal strength have been proven to be associated with GCC/GCIPL and/or pRNFL thickness; among these, age is considered to be the most significant determinant [11–14]. However, some of the previous studies investigated multiple potential determinants with limited analysis deep into each determinant, and some emphasized on one or two determinants. Thus, studies that are more comprehensive is needed to investigate which factors impact the OCT readings and how much the impacts are. The purpose of this study was to examine the influences of multiple demographic and ocular factors on the measurements of macular GCIPL thickness using Cirrus high-definition optical coherence tomography in a cohort of normal Chinese adults with detailed, comprehensive analyses.

## Methods

In this retrospective, observational study, all participants were consecutively recruited from the Zhongshan Ophthalmic Center, Sun Yat-sen University, Guangzhou, China, from February 2013 to January 2016. The study followed the tenets of the Declaration of Helsinki and was approved by the Institutional Review Board (IRB). Written informed consent was obtained from all participants after explanation of the nature and possible consequences of the study.

Eligibility was determined via demographic characteristics recording and a comprehensive ophthalmologic evaluation including measurement of uncorrected and best-corrected visual acuity, measurement of refractive error (cycloplegic refraction test for participants younger than 30-year-old, and manifest refraction test for participants of 30 years or older), slit lamp biomicroscopy examination, intraocular pressure (IOP) measurement using a Goldman applanation tonometer (Haag-Streit, Bern, Switzerland), angle evaluation using gonioscopy, dilated fundus examination, stereo disc photography (Kowa nonmyd a-D III; Kowa Optimed Inc., Aichi, Japan), visual field testing (Humphrey Visual Field Analyzer II; Carl Zeiss Meditec, Dublin, CA) using the Swedish Interactive Thresholding Algorithm (SITA) standard 24–2 program, and OCT scanning (Cirrus HD-OCT; Carl Zeiss Meditec, Dublin, CA).

For inclusion, the criteria were: (1) generally healthy individuals of age ≥ 18 years; (2) best-corrected visual acuity 20/20 or better; (3) a spherical equivalent (SE) refractive error between − 5.00 diopters (D) and + 1.00 D, astigmatism between − 1.00 D and + 1.00 D, and bilateral refractive difference of no more than 1.50 D in SE; (4) intraocular pressure < 21 mmHg; (5) normal and wide anterior chamber angle; (6) normal optic nerve head appearance (cup-to-disc ratio < 0.5 in either eye, with the asymmetry of ≤0.2, no evidence of optic disc hemorrhage or focal thinning of the rim); (7) reliable and normal visual field results which was defined as: mean deviation (MD) with a *P* value of > 5%, pattern standard deviation (PSD) with a *P* value of > 5%, no more than 3 adjacent points in the pattern deviation plot with a *P* value of < 5%, and glaucoma hemifield test within the normal range; besides, the test should have a false-positive error, a false-negative error, and a fixation loss of less than 15%, simultaneously. The appearance of the optic disc on stereoscopic fundus photographs and visual field results were evaluated independently by two glaucoma specialists (XX and HX) who were masked to all other information about the eyes. Inconsistencies between these two doctors were decided by a senior glaucoma expert (XL). The data of the eye would not be used if the three doctors did not reach an agreement on the classification.

The exclusion criteria were: (1) any known history of ocular disorders except mild or moderate refractive error; (2) history of ocular and/or brain trauma; (3) previous intraocular surgery; (4) optic media opacity; (5) medications usage or toxicosis that may cause IOP elevation or induce optic neuropathy; (6) neurological or systemic disorders that potentially affect visual field results and/or OCT readings, such as intracranial mass lesions, cerebrovascular diseases, neuromyelitis optica spectrum disorder, multiple sclerosis, radiation encephalopathy and optic neuropathy; (7) unsatisfactory image acquisition; (8) inability to cooperatively complete all examinations.

Participants were enrolled only if both eyes met the inclusion criteria. Both eyes were studied. Except for the comparisons of GCIPL thickness between right and left eyes, data from one randomly selected eye of each participant were used for analyses.

Eligible participants underwent central cornea thickness (CCT) measurement using Ultrasonic Pachymetry (DGH-1000, Storz Inc., Louis, MO, USA) and the axial length (AL) measurement using IOLMaster (Carl Zeiss Meditec, Dublin, CA, USA).

OCT images were obtained after eyes were dilated with tropicamide 1% and phenylephrine 2.5% (Mydrin®-P, Santen Pharmaceutical Co. Ltd., Osaka, Japan) using the same OCT device by a well-trained glaucoma specialist (XX). Macular Cube 200 × 200 scan (200 horizontal B-scans comprising 200 A-scans per B-scan within a cube measuring 6 × 6 × 2 mm centered on the fovea) and Optic Disc Cube 200 × 200 scan (200 horizontal B-scans comprising 200 A-scans per B-scan within a cube measuring 6 × 6 × 2 mm centered on the optic disc center) were performed respectively at the same visit. Artificial tear was provided if the participants complained dryness or discomfort. Images with signal strength of less than 6 and those with visible motion or blinking artifacts and obvious segmentation failure were discarded immediately followed by repetition(s) of the scan.

The distance between the inner boundary of the GCL and the outer boundary of the IPL yields the combined thickness of the GCL and IPL (termed “GCIPL”) by the ganglion cell analysis (GCA) algorithm. The GCIPL thickness were analyzed within an elliptical annulus area of 14.13 mm^2^ with a horizontal inner and outer radius of 0.6 and 2.4 mm, respectively; and a vertical inner and outer radius of 0.5 mm and 2.0 mm, respectively. The average, minimum, and 6 sectoral (superior, superonasal, inferonasal, inferior, inferotemporal, and superotemporal) GCIPL thickness parameters were calculated. The minimum GCIPL thickness was defined as the lowest measured value of the 1-degree intervals among all the 360 spokes. The average RNFL and quadrant (superior, temporal, inferior and nasal) circumpapillary RNFL thickness were calculated by the Cirrus analysis algorithm.

Participants were divided into 6 groups by age with an interval of 10 years. Participants were also divided into 2 groups based on refractive error: emmetropia group (SE between − 0.5D to + 1.0D) and myopia group (SE of <− 0.5D and ≥ − 5.0D).

Statistical analyses were performed using SPSS 25.0 (SPSS Inc., Chicago, IL, USA). Shapiro-Wilk test and Levene test were applied to test the normality and the homogeneity of variance, respectively. Comparisons of GCIPL thickness between different gender and refractive groups were performed using independent-samples *t*-test. Paired-samples *t*-test was used to evaluate the GCIPL thickness between the right eye and the left eye of each subject. Comparisons of the GCIPL thickness and refraction between multiple age groups were performed using one-way analysis of variance (ANOVA) with Bonferroni adjustment for pairwise comparisons. Correlations between GCIPL thickness and the following factors were analyzed using Pearson correlation coefficient: age, SE refractive error, IOP, CCT, AL, and OCT signal strength. Univariate linear regression analyses were performed to assess how age and the average pRNFL thickness affected GCIPL thickness, respectively. As age itself may impact pRNFL thickness and OCT signal strength, multicollinearity between age and pRNFL thickness, and between age and signal strength was diagnosed using variance inflation factor (VIF), respectively. A VIF of 1 means there is no multicollinearity in the regression model. A VIF above 4 indicates that multicollinearity might exist, and a VIF above 10 indicates that there is significant multicollinearity. A *P* value of < 0.05 was considered statistically significant. Except where stated otherwise, the data were presented as mean ± standard deviation (SD) values.

## Results

A total of 225 participants (96 males and 129 females) were enrolled. The mean age of the study population was 46.3 ± 16.5 years (range from 18 to 75 years of age). The average, minimum, superotemporal, superior, superonasal, inferonasal, inferior, and inferotemporal GCIPL thickness was 84.56 ± 5.36, 81.32 ± 5.58, 83.08 ± 5.37, 85.70 ± 5.95, 87.15 ± 6.26, 85.07 ± 6.11, 82.46 ± 5.76, and 83.88 ± 5.59 μm, respectively. Variability of GCIPL thickness were assessed upon the following possible determinants:

### Gender

The mean age of 96 males and 129 females was 45.9 ± 16.7 and 47.6 ± 16.8 years, respectively. Independent-samples t-test of the age difference between genders showed no statistical significance (*P* = 0.576). No statistically significant differences were found of average, minimum, and sectoral GCIPL thickness between genders (Table 1).

**Table 1:** Comparison of GCIPL Thickness (μm) between Males and Females

	Males	Females	*P*
Average	84.84 ± 5.21	84.33 ± 5.49	0.484
Minimum	82.04 ± 5.32	80.81 ± 5.69	0.100
Superotemporal	83.81 ± 5.18	82.53 ± 5.45	0.076
Superior	85.90 ± 5.69	85.61 ± 6.08	0.714
Superonasal	87.18 ± 6.46	86.97 ± 5.99	0.797
Inferonasal	84.91 ± 6.03	85.07 ± 6.08	0.842
Inferior	82.58 ± 5.34	82.43 ± 6.03	0.840
Inferotemporal	84.67 ± 5.66	83.32 ± 5.42	0.069

### Laterality

Table 2 presented the comparisons of the variability between right and left eyes of all subjects, showing qualitatively similar GCIPL thickness in fellow eyes except that of the superonasal sector, where the left eyes yielded significant thicker GCIPL than the right eyes.

**Table 2:** Comparison of GCIPL Thickness (μm) between Right Eyes and Left Eyes

	OD	OS	Differences^a^	*P*
Average	84.40 ± 5.53	84.70 ± 5.30	− 0.295 ± 2.139	0.160
Minimum	81.25 ± 6.04	81.49 ± 5.07	−0.238 ± 4.349	0.576
Superotemporal	83.01 ± 5.56	83.31 ± 5.05	−0.305 ± 3.010	0.302
Superior	85.56 ± 6.12	85.95 ± 5.81	−0.390 ± 2.963	0.180
Superonasal	86.45 ± 6.39	87.71 ± 6.04	−1.267 ± 3.256	**< 0.001**
Inferonasal	85.09 ± 6.34	84.81 ± 5.81	0.276 ± 3.857	0.465
Inferior	82.50 ± 5.80	82.41 ± 5.83	0.086 ± 3.117	0.779
Inferotemporal	83.88 ± 5.69	83.83 ± 5.45	0.048 ± 3.093	0.875

*OD* Right eyes, *OS* Left eyes. ^a^Difference: the measured value of the right eye minus the measured value of the left eye

### Age

The grouping of six age groups, and the GCIPL measurements of each group with group-wise comparison results were presented in Table 3. GCIPL thickness increased slowly with age in younger adults and reached its peak at 40–49 years of age. Thereafter, it decreased rapidly with age. Pairwise comparisons showed statistically significant difference in each GCIPL parameter between Group 4 and Group 5, and between Group 4 and Group 6. Given that the refractive error may have potential impacts on GCIPL thickness independent of other factors, refraction status of each age group was evaluated. The SE from Group 1 to 6 was (− 2.89 ± 1.83) D, (− 0.11 ± 1.67) D, (0.45 ± 0.54) D, (− 0.20 ± 0.95) D, (− 0.19 ± 1.03) D, and (0.70 ± 1.79) D. One-way ANOVA showed statistically significant difference among the 6 groups (*P* < 0.001). Statistically significant differences in SE was found in further pairwise comparisons between Group 1 and other 5 groups (all *P* < 0.001), whereas no significant difference was found between Group 2 to 6, respectively (*P* = 0.083–0.969).

**Table 3:** Measurements (μm) and Comparisons of GCIPL Thickness between Six Age Groups

Group	Age (years)	Average	Minimum	Supero-temporal	Superior	Superonasal	Inferonasal	Inferior	Infero-temporal
1: 18–29 years (*n* = 48)	24.0 ± 3.5	84.69 ± 4.56	82.13 ± 5.39	83.25 ± 4.78	86.44 ± 4.74	87.17 ± 5.46	85.19 ± 4.82	82.52 ± 4.58	83.81 ± 4.84
2: 30–39 years (*n* = 38)	34.3 ± 3.0	84.87 ± 5.86	82.03 ± 5.15	83.82 ± 5.43	85.84 ± 6.20	87.58 ± 7.44	85.32 ± 6.98	82.68 ± 6.24	84.21 ± 5.46
3: 40–49 years (*n* = 32)	43.6 ± 3.0	87.25 ± 5.94	83.31 ± 5.92	84.84 ± 5.47	88.72 ± 6.73	89.75 ± 6.92	88.16 ± 6.12	85.84 ± 6.46	86.06 ± 5.63
4: 50–59 years (*n* = 45)	55.4 ± 2.3	85.20 ± 3.84	81.56 ± 4.59	83.31 ± 5.42	86.53 ± 4.50	88.07 ± 3.80	86.00 ± 4.56	82.62 ± 3.98	84.47 ± 4.73
5: 60–69 years (*n* = 44)	63.9 ± 2.5	83.55 ± 4.97	80.34 ± 5.26	82.16 ± 5.20	83.95 ± 5.30	85.91 ± 5.60	83.77 ± 5.54	81.68 ± 5.49	83.52 ± 5.46
6: 70–79 years (*n* = 18)	72.6 ± 2.8	79.47 ± 6.32	76.06 ± 6.49	79.71 ± 5.68	80.59 ± 7.47	81.18 ± 6.61	78.47 ± 6.67	77.59 ± 7.20	79.18 ± 7.47
Statistics									
P*		**< 0.001**	**< 0.001**	**0.029**	**< 0.001**	**< 0.001**	**< 0.001**	**0.003**	**< 0.001**
1 vs 2		0.871	0.932	0.622	0.626	0.748	0.917	0.891	0.734
1 vs 3		**0.029**	0.331	0.187	0.076	0.057	**0.023**	**0.008**	0.069
1 vs 4		0.629	0.608	0.956	0.934	0.464	0.490	0.929	0.560
1 vs 5		0.285	0.111	0.323	**0.035**	0.309	0.233	0.464	0.797
1 vs 6		**< 0.001**	**< 0.001**	**0.018**	**< 0.001**	**< 0.001**	**< 0.001**	**0.002**	**0.003**
2 vs 3		0.053	0.317	0.417	**0.034**	0.127	**0.038**	**0.017**	0.154
2 vs 4		0.769	0.690	0.664	0.577	0.708	0.584	0.959	0.830
2 vs 5		0.244	0.156	0.157	0.130	0.204	0.220	0.410	0.565
2 vs 6		**< 0.001**	**< 0.001**	**0.008**	**0.002**	**< 0.001**	**< 0.001**	**0.002**	**0.002**
3 vs 4		0.084	0.157	0.210	0.094	0.220	0.101	**0.012**	0.202
3 vs 5		**0.002**	**0.018**	**0.029**	**< 0.001**	**0.006**	**0.001**	**0.001**	**0.044**
3 vs 6		**< 0.001**	**< 0.001**	**0.001**	**< 0.001**	**< 0.001**	**< 0.001**	**< 0.001**	**< 0.001**
4 vs 5		0.128	0.285	0.304	**0.031**	0.087	0.065	0.419	0.410
4 vs 6		**< 0.001**	**< 0.001**	**0.017**	**< 0.001**	**< 0.001**	**< 0.001**	**0.001**	**0.001**
5 vs 6		**0.006**	**0.005**	0.105	**0.037**	**0.006**	**0.001**	**0.010**	**0.005**

*: *P* values are statistic results for general comparisons of the GCIPL thickness parameters among the six age groups using one-way ANOVA. *P* values for post-hoc pairwise comparisons between each age group are presented following the results of general comparisons

**Fig. 1:**
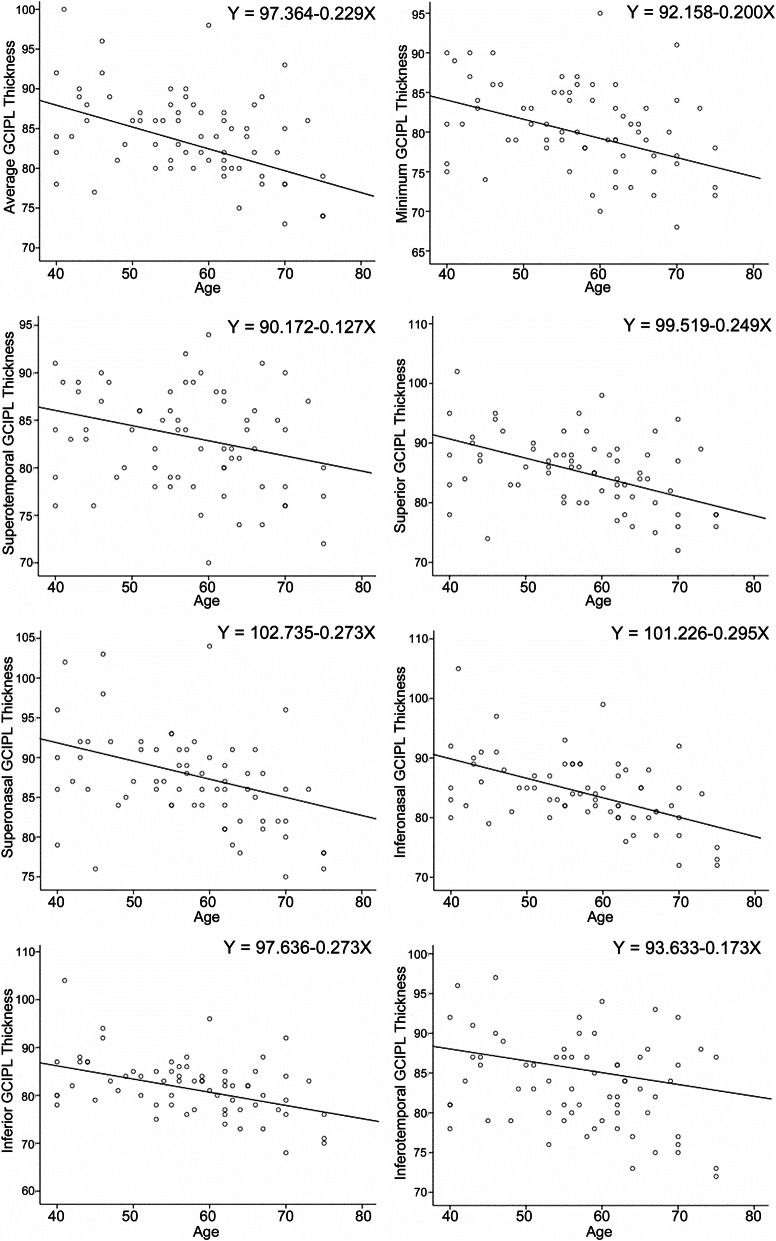
The scatterplots and the regression equations between GCIPL thickness parameters and age in subjects of 40 years or older

There was a negative correlation between GCIPL thickness and age. Statistical significance was found in all analyses except that between the inferotemporal GCIPL thickness and age. Significant negative correlation of GCIPL thickness and age was found in all GCIPL parameters but superotemporal GCIPL thickness in subjects of 40 years or older (Table 4). Regression analyses performed in 139 subjects older than 40 years showed that GCIPL thickness parameters were predictable by age (Fig. 1). For example, average GCIPL thickness decreased by 0.229 μm for every 1 year increase in age.

**Table 4:** Correlation Analyses between GCIPL Thickness and Age

	All subjects (*n* = 225)		Subjects of 40 years or older (*n* = 139)	
	*r*	*P*	*r*	*P*
Average	−0.175	**0.009**	−0.402	**< 0.001**
Minimum	−0.217	**0.001**	−0.339	**< 0.001**
Superotemporal	−0.157	**0.019**	− 0.281	**0.001**
Superior	− 0.215	**0.001**	− 0.420	**< 0.001**
Superonasal	−0.168	**0.012**	−0.410	**< 0.001**
Inferonasal	−0.187	**0.005**	−0.444	**< 0.001**
Inferior	−0.151	**0.024**	−0.374	**< 0.001**
Inferotemporal	−0.119	0.075	−0.293	**< 0.001**

### IOP, AL, and CCT

Mean IOP was (14.3 ± 2.5) mm Hg (ranging from 9 to 19 mmHg). Mean AL was (23.84 ± 0.85) mm (ranging from 22.12 to 26.24 mm). Mean CCT was (554.1 ± 27.1) μm (ranging from 497 to 628 μm). No statistically significant correlation was found between each GCIPL parameter with IOP, AL, or CCT (Table 5).

**Table 5:** Correlation between GCIPL Thickness and Intraocular Pressure, Axial Length, Central Cornea Thickness, and Spherical Equivalent Refractive Error

	IOP		AL		CCT		SE	
	*r*	*P*	*r*	*P*	*r*	*P*	*r*	*P*
Average	0.050	0.617	−0.096	0.339	0.065	0.516	0.107	0.285
Minimum	−0.027	0.790	−0.074	0.459	−0.046	0.645	0.039	0.696
Superotemporal	0.055	0.582	−0.078	0.435	−0.011	0.912	0.072	0.474
Superior	0.037	0.715	−0.004	0.967	0.086	0.390	0.070	0.486
Superonasal	0.062	0.536	−0.059	0.555	0.079	0.429	0.105	0.292
Inferonasal	0.015	0.880	−0.147	0.141	0.083	0.407	0.149	0.135
Inferior	0.046	0.644	−0.146	0.144	0.086	0.392	0.124	0.213
Inferotemporal	0.058	0.565	−0.098	0.327	−0.004	0.968	0.066	0.511

*IOP* Intraocular pressure, *AL* Axial length, *CCT* Central cornea thickness, *SE* Spherical equivalent refractive error

### Refractive error

Of all the 225 eyes enrolled in this study, mean SE refractive error was (− 0.57 ± 1.88) D, ranging from − 5.00D to + 1.00D. No statistically significant correlation was found between each GCIPL parameter and SE (Table 5, last 2 columns). All participants were also divided into emmetropic group (122 eyes, mean SE of 0.60 ± 0.85 D, mean age of 48.5 ± 6.6 years) and myopic group (103 eyes, mean SE of − 2.38 ± 1.57 D, mean age of 42.7 ± 9.4 years). No statistically significant difference in age was found between the two groups (*P* = 0.181). Statistically significant differences were found in inferonasal and inferior GCIPL thickness only (*P* = 0.008 and 0.024, respectively; Table 6).

**Table 6:** Comparison of GCIPL Thickness (μm) between Emmetropic and Myopic Eyes

	Emmetropia	Myopia	*P*
Average	85.11 ± 6.53	83.10 ± 4.01	0.056
Minimum	82.16 ± 5.99	80.43 ± 4.81	0.127
Superotemporal	83.26 ± 5.86	82.08 ± 4.05	0.231
Superior	86.26 ± 6.81	84.18 ± 4.66	0.070
Superonasal	87.81 ± 7.52	85.83 ± 4.44	0.980
Inferonasal	86.18 ± 7.10	83.00 ± 4.81	**0.008**
Inferior	83.52 ± 6.95	80.88 ± 4.68	**0.024**
Inferotemporal	83.84 ± 6.64	82.43 ± 4.43	0.238

### RNFL thickness

**Fig. 2:**
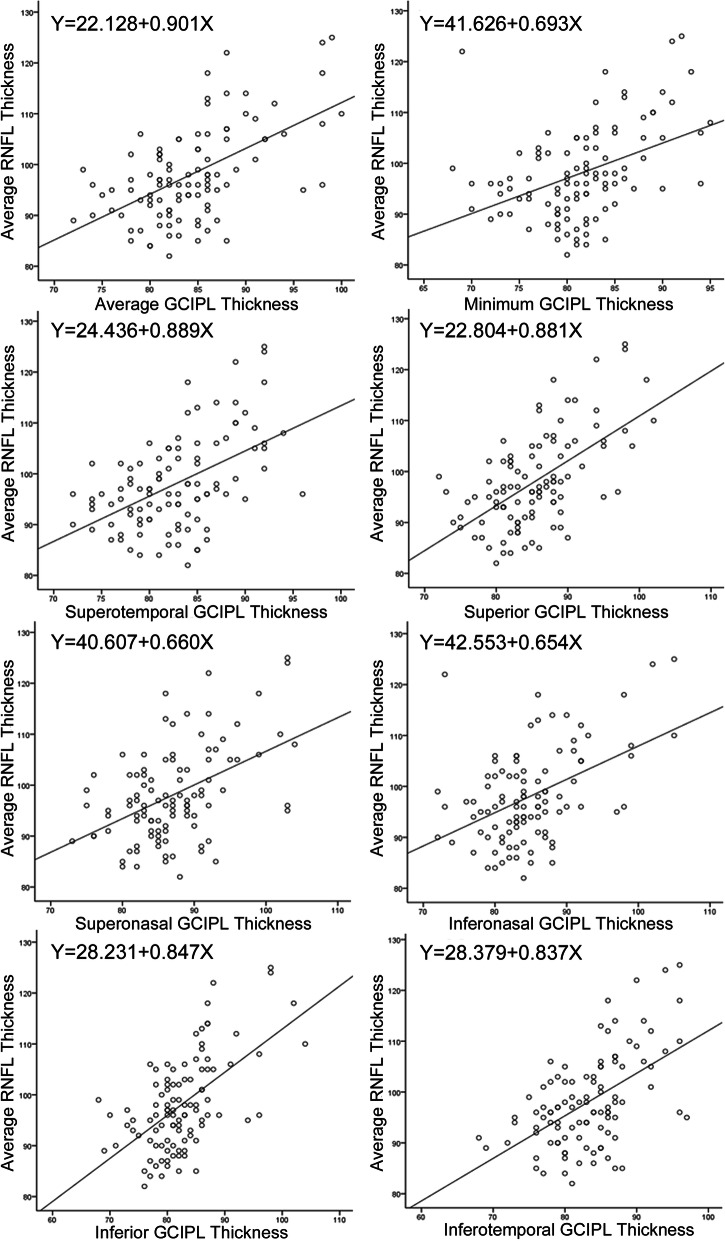
The scatterplots and the regression equations between GCIPL thickness parameters and the average RNFL thickness

The mean average, superior, temporal, inferior, and nasal RNFL thickness was (98.08 ± 9.17) μm (range: 82 to 125 μm), (123.99 ± 15.42) μm (range: 94 to 187 μm), (71.83 ± 10.76) μm (range: 55 to 102 μm), (130.17 ± 16.65) μm (range: 102 to 191 μm), and (66.25 ± 10.08) μm (range 42 to 95 μm), respectively. Statistically significant positive correlation was found between all GCIPL thickness parameters and average RNFL thickness (all *P* < 0.001). More correlation analyses were conducted between each GCIPL thickness parameter and the superior, temporal, inferior, and nasal RNFL thickness, respectively (Table 7). Regression analyses showed that mean RNFL thickness was predictable by GCIPL thickness parameters (Fig. 2). For example, the average RNFL thickness decreased by 0.901 μm for every 1 μm decrease in average GCIPL thickness. Further analysis of possible impacts of age on average RNFL thickness was performed using multivariate linear regression model. Multicollinearity diagnosis showed that the VIF was 1.053 in this model, indicating no significant multicollinearity was found.

**Table 7:** Correlation Analyses between GCIPL Thickness and RNFL Thickness Parameters

RNFL	Average		Superior		Temporal		Inferior		Nasal	
GCIPL	*r*	*P*	*r*	*P*	*r*	*P*	*r*	*P*	*r*	*P*
Average	0.563	**< 0.001**	0.413	**< 0.001**	0.203	**0.040**	0.540	**< 0.001**	0.332	**0.001**
Minimum	0.423	**< 0.001**	0.280	**0.004**	0.202	**0.042**	0.399	**< 0.001**	0.251	**0.011**
Supero-temporal	0.508	**< 0.001**	0.362	**< 0.001**	0.223	**0.024**	0.463	**< 0.001**	0.312	**0.001**
Superior	0.588	**< 0.001**	0.447	**< 0.001**	0.225	**0.023**	0.520	**< 0.001**	0.373	**< 0.001**
Supero-nasal	0.471	**< 0.001**	0.341	**< 0.001**	0.156	0.117	0.448	**< 0.001**	0.302	**0.002**
Infero-nasal	0.461	**< 0.001**	0.328	**0.001**	0.124	0.215	0.479	**< 0.001**	0.264	**0.007**
Inferior	0.579	**< 0.001**	0.443	**< 0.001**	0.188	0.058	0.582	**< 0.001**	0.292	**0.003**
Infero-temporal	0.537	**< 0.001**	0.395	**< 0.001**	0.238	**0.016**	0.492	**< 0.001**	0.312	**0.001**

*GCIPL* Ganglion cell-inner plexiform layer, *RNFL* Peripapillary retinal nerve fibre layer

### OCT signal strength

The average signal strength of macular cube 200 × 200 scanning protocol was (8.40 ± 1.22). Based on that, the correlation between the signal strength and the GCIPL thickness was analyzed and statistically significant positive correlation was found in all GCIPL thickness parameters (Table 8). Further analysis of possible impacts of age on signal strength was performed using multivariate linear regression model. Multicollinearity diagnosis showed that the VIF was 1.029 in this model, indicating no significant multicollinearity was found.

**Table 8:** Correlation Analyses between GCIPL Thickness and OCT Signal Strength Using Macular Cube 200 × 200 Scanning Protocol

	GCIPL Thickness (μm)	*r*	*P*
Average	84.44 ± 5.64	0.297	**< 0.001**
Minimum	81.57 ± 5.81	0.344	**< 0.001**
Superotemporal	82.73 ± 6.31	0.365	**< 0.001**
Superior	85.43 ± 5.96	0.350	**< 0.001**
Superonasal	86.93 ± 6.41	0.319	**< 0.001**
Inferonasal	85.33 ± 6.21	0.234	**< 0.001**
Inferior	82.76 ± 6.32	0.395	**< 0.001**
Inferotemporal	83.52 ± 5.87	0.341	**< 0.001**

## Discussion

In this study, we evaluated multiple determinants of macular GCIPL thickness in normal Chinese adults and demonstrated that GCIPL thinning was associated with older age, thinner pRNFL, and weaker OCT scanning signal strength. In general, gender, laterality, refractive status (when the refractive error is between +1D and -5D), IOP, CCT, and AL had no significant impacts on macular GCIPL thickness.

Age was one of the most significant impact factors in determining macular GCIPL thickness. In this study, we found that the overall trend of GCIPL thickness changing with age was as follows: GCIPL thickness increased slowly with age in younger adults; after reaching the peak at 40–49 years of age, it decreased rapidly with age, which was consistent with the findings of Mwanza et al. [14] However, previous studies involving Asian populations showed that pRNFL thickness was comparatively thicker in teenagers and reached its peak at 20–29 years of age, then gradually became thinner [15, 16]. These findings suggested that GCIPL and RNFL thickness changes may not necessarily be synchronized.

We found that for each additional year over 40 years of age, average, minimum, superotemporal, superior, superonasal, inferonasal, inferior, and inferotemporal GCIPL thickness decreased by 0.229, 0.200, 0.127, 0.249, 0.273, 0.295, 0.273, and 0.173 μm, respectively. Animal experiments demonstrated that the age-related change of RGC predominately manifested as axon loss, while the RGC cell count is relatively constant [17]. As the retina expands with age while the total cell counts retain, the RGC density decreases. The phenomenon of retina expansion with age was also found in human eyes, but the main difference with the animal eyes was that the number of RGC soma also declined with age in human [18]. Therefore, it is explainable that the GCIPL gets thinner with age in an OCT-based thickness evaluation, as shown in this study. It has been proven by multiple studies that GCC or GCIPL thickness decreased with age, even though the age-related RGC loss rate varies. Annual RGC loss rate was between 0.07 to 0.61%, as reported in studies with the sample size between 12 eyes to 72 eyes [19–23]. In this study, we further approved that the age-dependent GCIPL thickness change was nonlinear with age. However, this age-related variability of the OCT measurements may not be completely attributed to inter-subject variability, which is considerably significant even in normal populations. When ganglion cells reduce with age, the migrant amacrine cells and other non-neuronal components may partially compensate the space which is previously predominated by ganglion cells. As such, the actual cell loss may be masqueraded and the age-RGC loss correlation may become more unpredictable. Moreover, ganglion cells layer in the macular region, making it more complicated to evaluate the defined pattern of region- and eccentricity-associated, age-dependent RGC loss.

When evaluating the potential causative impacts of axial length on GCIPL thickness, contradictory conclusions were drawn, which was similar to RNFL thickness. Some studies proposed that the GCIPL and RNFL thickness were negatively correlated to axial length [24–27]. Studies with a larger sample size and/or a wider range of refractive status, generally indicated that only less than 0.5% GCIPL thickness change was attributed to per millimeter axial length change [13, 14]. Such minor changes could hardly reflect any practical clinical significance. Another reason that axial length may have some impacts on the GCIPL thickness measurement, but not necessarily the actual anatomic cell counts may be ascribed to the optical effects. Since the Cirrus OCT model eye adopts a calibrated value of 24.46 mm as the default axial length setting with a fixed measuring angular distance of approximately 12°, the actual scanning area would be larger than the “standard” retinal area due to the optical magnification effect in eyes longer than 24.46 mm. As the macular ganglion cell counts drop dramatically as the eccentricity increases outward from the foveal center, average ganglion cell estimates or GCIPL thickness in these eyes may therefore be underestimated. On the contrary, in eyes shorter than 24.46 mm, the actual scanning area is smaller than the preset area where the ganglion cells are more crowded and thus a thicker GCIPL measurement may be falsely generated.

Other studies declined the direct impacts of axial length on RGC growth or apoptosis and claimed that axial length had no significant correlation with macular GCIPL thickness [28, 29], which our findings was consistent with. The relatively small sample size could be one possible cause. Also, our inclusive criteria for spherical equivalent refractive error were − 5.00 D and + 1.00 D. The exclusion of highly myopic eyes restricted the variability of axial length, thus minimizing the interference of magnification effects on location of the retinal area being scanned that could potentially influence the GCIPL thickness measurements. Similarly, no significant association between refractive error and GCIPL thickness was found in our study. The difference of average, minimum, and most sectoral GCIPL thickness between the two refractive groups were not statistically significant, except that in inferonasal and inferior sectors. Histological studies of both human and animals have found that the RGC were denser nasally than temporally, and superiorly than inferiorly, with distinct inter-subject variability, which might have indicated the discrepant anatomic distribution pattern in emmetropic and myopic eyes [30]. The thickest GCIPL was detected in the superonasal sector, where no significant difference in emmetropic and myopic groups was found, suggesting that the density in this sector may have partially offset the difference caused by refractive error and/or axial length. This sector may have poorer performance in diagnosing glaucoma due to its least glaucomatous susceptibility.

In a cohort of participants that no significant correlation between age and pRNFL thickness was found, the finding that pRNFL thickness had statistically significant positive correlation with macular GCIPL thickness was not surprising and was consistent with previous studies [14, 31]. The axon and soma of the ganglion cells are closely related cellular components, and both can be remarkably affected by glaucoma. Thus, GCIPL and pRNFL thickness are both important and sensitive for early detection of glaucoma. The regression analysis showed that the average RNFL thickness decreased by 0.901 μm for every 1 μm decrease in average GCIPL thickness. Overk et al. [32] found that lesions in the axon may occur earlier than in the soma in some neurodegenerative diseases such as Alzheimer’s disease and Parkinson’s disease, indicating a ‘reverse’ pathogenesis pathway for the primary causative factor of glaucoma. In the pathophysiological development process of glaucoma, whether GCIPL is affected primarily and causes changes in pRNFL, or vice versa, still needs further investigations.

The stronger the OCT signal strength, the deeper retinal tissue the light achieved. As the reflection of the boundaries got enhanced, the segmentation of each layer was more accurate. We found that OCT signal strength was positively correlated to macular GCIPL thickness. Likewise, the signal strength of Cirrus OCT was found to be positively correlated with RNFL thickness [33]. Even though age and OCT signal strength was not correlated in our study, the impact of age on OCT signal strength was notable in clinical practice. Attempts for reaching higher OCT signal strength are recommended to minimize possible underestimate of GCIPL thickness, especially in older populations.

There were several limitations of this study. First, it was a cross-sectional, retrospective study with a comparatively small sample size. Analyses based on a larger sample size is essential to provide more reliable evidence in standardizing clinical interpretation. Second, the subjects were not strictly matched. For instance, the refractive error in the first age group differed significantly with other 5 age groups, while the age of the emmetropic group and the myopic group differed, as well. Third, only ocular predictors were evaluated. Systemic predictors such as history of diabetes, cigarette smoking history, blood pressure, serum lipid levels should be taken into account. More comprehensive investigations are expected in future studies.

## Conclusion

In conclusion, the GCIPL thickness measured by Cirrus OCT in normal Chinese subjects was associated with age, RNFL thickness, and signal strength of OCT scanning. No significant association was found between GCIPL thickness and gender, laterality, refractive status, intraocular pressure, axial length, and central corneal thickness. These should be considered when making clinical interpretation of GCIPL thickness.

## Data Availability

The datasets used and/or analysed during the current study are available from the corresponding author on reasonable request.

## Abbreviations

GCIPL: Ganglion cell-inner plexiform layer
OCT: Coherence tomography
SE: Spherical equivalent
IOP: Intraocular pressure
AL: Axial length
CCT: Central cornea thickness
pRNFL: Peripapillary retinal nerve fibre layer
RGC: Retinal ganglion cell
ONH: Optic nerve head
GCL: Ganglion cell layer
GCC: Ganglion cell complex
MD: Mean deviation
PSD: Pattern standard deviation
